# The Triage Frailty and Comorbidity Tool for Risk Stratification of Older Patients Admitted from the Emergency Department to a Geriatric Ward

**DOI:** 10.3390/healthcare14213832

**Published:** 2026-11-09

**Authors:** Arian Zaboli, Gianni Turcato, Christine Kirchlechner, Ferisa C. Sleghel, Donatella Bertagnolli, Marta Ziller, Francesco Brigo, Marianne Siller, Ingrid Ruffini

**Affiliations:** 1 Health Professions Management, South Tyrolean Health Authority (SABES-ASDAA), 39100 Bolzano, Italy; 2 Department of Internal Medicine, Intermediate Care Unit, Hospital Alto Vicentino (AULSS-7), 36014 Santorso, Italy; gianni.turcato@yahoo.it; 3 Department of Geriatrics, Hospital Meran (SABES-ASDAA), 39012 Merano, Italy; 4 Emergency Department, Hospital of Merano (SABES-ASDAA), 39012 Merano, Italy; 5 Innovation, Research and Teaching Service (SABES-ASDAA), Teaching Hospital of the Paracelsus Medical Private University (PMU), 39100 Bolzano, Italy

**Keywords:** triage frailty and comorbidity tool, frailty, emergency service, hospital, triage, aged, geriatric assessment, risk assessment, mortality

## Abstract

**Introduction**: Frailty assessment in the Emergency Department (ED) may provide prognostic information beyond triage, but the value of the Triage Frailty and Comorbidity Tool (TFC) in older patients admitted to geriatric wards is uncertain. **Methods:** This retrospective study included patients aged ≥75 years admitted directly from the ED to Merano Hospital Geriatric Ward between May and September 2025. The primary outcome was 90-day mortality; in-hospital mortality was secondary. Analyses included logistic regression, AUROC estimation, predefined TFC ≥ 10% threshold performance, and Kaplan–Meier curves. **Results:** A total of 225 patients were included. Median age was 86 years (IQR 82–90), median length of stay was 9 days (IQR 6–13), 90-day mortality was 27.6% (62/225), and in-hospital mortality was 11.5% (26/225). Patients who died within 90 days had higher TFC values than survivors [49.5 (31.4–72.3) vs. 19.4 (6.5–43.8); *p* < 0.001]. Each 10-point increase in TFC was associated with higher odds of 90-day mortality (OR 1.43, 95% CI 1.26–1.63; *p* < 0.001) and in-hospital mortality (OR 1.38, 95% CI 1.18–1.62; *p* < 0.001). The AUROC was 0.747 for 90-day mortality and 0.732 for in-hospital mortality. TFC ≥ 10% identified 54/62 deaths, with a sensitivity of 87.1% and NPV of 87.9%. Survival was lower in patients with TFC ≥ 10% (log-rank *p* = 0.001). **Conclusions:** The TFC showed prognostic value in older patients admitted from the ED to geriatric care. Although discrimination was moderate, the TFC may support risk stratification during ED-to-ward transition.

## 1. Introduction

In recent years, increasing attention has been paid to the early identification of frailty among patients attending the Emergency Department (ED) [1,2]. This issue is particularly relevant in older patients, in whom the assessment of clinical urgency alone may not be sufficient to describe overall vulnerability, reduced functional reserve, and the risk of adverse outcomes after ED admission [1,2]. However, there is still no universally accepted approach for frailty assessment in this setting [2,3]. Some tools, such as the Clinical Frailty Scale (CFS), were mainly developed for older populations and require a broader clinical and functional assessment [3,4]. Others, such as the Triage Frailty and Comorbidity Tool (TFC), were specifically designed for the triage and ED setting, with the aim of rapidly integrating information on comorbidity, functional autonomy, cognitive status, mobility, and recent hospitalizations [5,6].

Frailty assessment in the ED has often been investigated in relation to its ability to identify vulnerable patients early, improve risk stratification, and support more appropriate care pathways [5,6]. However, it remains uncertain whether information collected at the time of ED access retains prognostic value after hospital admission, particularly among older patients admitted to geriatric wards. This population represents a clinically and care-intensive group, in which comorbidity, functional dependency, cognitive impairment, and impaired mobility may substantially contribute to the risk of in-hospital and short-term mortality [7].

A further relevant issue is that patients requiring hospital admission after ED evaluation may experience variable waiting times before transfer to an inpatient ward, often due to organizational or structural factors [8,9]. During this phase, the availability of simple and early indicators of frailty and comorbidity could help identify patients at higher clinical risk and support more accurate prognostic stratification during the transition from the ED to the inpatient setting. Although previous studies have evaluated the TFC at presentation in the general ED population, its prognostic performance among patients subsequently admitted to geriatric care remains uncertain [5,6]. This selected population consists exclusively of older patients, in whom frailty and comorbidity are already highly prevalent. Whether the TFC can distinguish different levels of mortality risk within this population is therefore a distinct question, relevant to the continuity of prognostic information from the ED to the inpatient ward.

Therefore, this study aimed to evaluate the prognostic performance of the TFC, assessed during ED evaluation, in patients aged 75 years or older subsequently admitted directly to a geriatric ward. Specifically, we assessed whether the TFC could identify a subgroup at increased risk of 90-day mortality, the primary outcome, and in-hospital mortality, the secondary outcome. This analysis may help clarify whether a tool developed for the ED setting retains prognostic usefulness during the subsequent phase of geriatric hospitalization.

## 2. Methods

### 2.1. Study Design and Setting

This was a retrospective observational study conducted from 1 May 2025 to 30 September 2025 at the Geriatric Ward of Merano Hospital, South Tyrol, Italy.

The Geriatric Ward has 30 ordinary inpatient beds and represents one of the two dedicated geriatric wards in the Province of South Tyrol.

### 2.2. Patients

All patients admitted directly from the ED to the Geriatric Ward during the study period were screened for eligibility. Patients were included if they were aged 75 years or older and had an available TFC assessment from the ED. Patients admitted from other hospital wards or healthcare facilities, or temporarily hosted in the ward while formally assigned to another specialty, were excluded. This population was selected to assess whether TFC information collected at ED presentation retains prognostic value after admission to geriatric care, in a setting characterized by advanced age and a high burden of frailty and multimorbidity.

Three geriatricians manually reviewed the electronic medical records of eligible patients and collected the study variables using a data collection form developed for the study. Data extraction and TFC verification were completed before registry-based ascertainment of 90-day vital status. In-hospital deaths were visible in the medical records; the physicians were therefore not blinded to in-hospital mortality during verification.

### 2.3. Variables

The following demographic and clinical variables were collected: sex, categorized as female or male; age in years; date of admission from the ED; and previous medical history. All comorbidities required to calculate the Charlson Comorbidity Index (CCI) were retrieved from the medical records [10].

The TFC was originally developed to estimate 90-day mortality among adults attending the ED, without restricting its application to geriatric patients. It was intended to identify vulnerability related to comorbidity and frailty across adult age groups, including among patients classified as non-urgent at triage [5,6,11].

The TFC score and all its individual components were recorded [11]. These included the CCI, mode of arrival to the ED, functional autonomy at home, chronic motor impairment, chronic cognitive impairment, and hospitalization within the previous 90 days [11]. Mode of arrival was categorized as self-arrival or arrival by emergency medical services. Functional autonomy was defined according to whether the patient was autonomous in activities at home before admission. Motor status was categorized into three levels: no impairment, use of walking aids, or bedridden status. Cognitive status was categorized into three levels: no cognitive impairment, mild cognitive impairment, or severe cognitive impairment. Mild cognitive impairment was defined as the presence of cognitive disease with preserved ability to communicate independently, whereas severe cognitive impairment was defined as the inability to communicate independently [11].

TFC assessment is mandatory as part of routine ED assessment, and a score was available for all the included patients. No patient was excluded because of a missing TFC assessment. The recorded score and its individual components were cross-checked against the available clinical documentation, and the calculation was verified using the original TFC formula. This verification focused on medical history and premorbid functional status, which could have been incompletely documented at ED presentation when patients had communication difficulties or relatives were unavailable. Where necessary, component data were corrected and the score was recalculated before analysis.

Verification was not restricted to information documented at ED presentation. Inpatient medical records were also reviewed to clarify the patient’s medical history and premorbid functional status.

Hospital length of stay was calculated from the date of ward admission to the date of discharge. Discharge status at the end of the index hospitalization was also recorded and categorized as discharge home, return to a long-term care facility where the patient was already resident, new admission to a long-term care facility, transfer to a post-acute care facility, or death during hospitalization. In-hospital mortality and 90-day mortality were verified through the hospital registry office.

### 2.4. Outcomes

The primary outcome was 90-day mortality occurring during hospitalization and after discharge.

Follow-up started at ED presentation. Ninety-day mortality included all deaths occurring within ninety days of this date, both during the index hospitalization and after discharge. Vital status at 90 days was verified through the hospital registry office for all the patients, with complete outcome ascertainment and no missing mortality outcomes.

This outcome was selected because medium-term mortality is commonly used in studies evaluating frailty and vulnerability in older patients, although no single gold-standard outcome exists for the assessment of frailty.

The secondary outcome was in-hospital mortality, defined as death occurring during the index hospital stay. Mortality data were obtained from the registry office.

### 2.5. Sample Size

The sample size was justified on the basis of the primary outcome, 90-day mortality. Based on previous studies in acutely hospitalized older patients and acute geriatric ward populations, reporting short-term mortality rates ranging from approximately 20% to more than 30%, an expected 90-day mortality rate of approximately 25–30% was considered plausible for this selected cohort of older patients admitted to a geriatric ward after ED access [12,13].

Assuming this expected event rate, a sample size of at least 200 patients was expected to provide approximately 50–60 deaths within 90 days. This number of events was considered adequate for the planned analyses, which focused on the prognostic performance of a single composite score rather than on the development of a multivariable prediction model.

For the predefined TFC threshold of ≥10%, assuming an expected sensitivity of approximately 85% for 90-day mortality, at least 49 events would be required to estimate sensitivity with a precision of approximately ±10%. With an expected mortality rate of 25%, this corresponds to approximately 196 patients. In addition, for ROC analysis, assuming an expected AUROC of approximately 0.75, a sample of 200 patients with a 25–30% event rate would provide an estimated standard error of approximately 0.04 and a 95% confidence interval half-width of approximately 0.08 around the AUROC estimate.

Therefore, a target sample size of at least 200 patients was considered adequate for the primary objective of this study. The final study population included 225 patients, with 62 deaths within 90 days, exceeding the minimum number of expected events required for the planned performance analyses.

### 2.6. Statistical Analysis

Statistical analysis was performed using STATA version 16.2 (StataCorp, College Station, TX, USA). Continuous variables were reported as median and interquartile range (IQR), while categorical variables were reported as absolute numbers and percentages. The distribution of continuous variables was assessed before analysis. Comparisons between patients alive and dead at 90 days were performed using the Mann–Whitney U test for continuous variables and the chi-square test or Fisher’s exact test, as appropriate, for categorical variables. A *p*-value < 0.05 was considered statistically significant.

The association between TFC and mortality outcomes was first assessed using univariable logistic regression models. TFC was analyzed as a continuous variable, and odds ratios were reported for each 10-percentage-point increase in TFC. Predicted probabilities of 90-day mortality and in-hospital mortality across the full range of TFC values were estimated from the logistic regression models and graphically represented.

Discrimination for 90-day and in-hospital mortality was assessed using the area under the receiver operating characteristic curve (AUROC), with 95% confidence intervals (95% CI) estimated using DeLong’s variance estimator. The AUROCs of TFC, the age-adjusted CCI alone, and chronological age alone were compared using DeLong’s test for correlated ROC curves, as all three indicators were evaluated in the same patients. Holm correction was applied to the three pairwise comparisons within each mortality outcome.

The predefined TFC threshold of ≥10% was retained from previous validation work [11]. Sensitivity, specificity, positive predictive value (PPV), and negative predictive value (NPV) were calculated for 90-day mortality and, as an exploratory analysis, for in-hospital mortality. Corresponding 95% confidence intervals were calculated using the exact binomial Clopper–Pearson method. The association between TFC ≥ 10% and 90-day mortality was also assessed using univariable logistic regression.

Calibration for 90-day mortality was assessed using the probabilities calculated with the original TFC formula. Mean predicted risk was compared with observed mortality. Calibration-in-the-large was estimated using an intercept-only logistic regression with the logit of the predicted probability included as an offset. The calibration slope was estimated using logistic regression with this logit as the predictor and an unconstrained intercept. Ideal values were 0 for the calibration intercept and 1 for the calibration slope. Overall predictive accuracy was evaluated using the Brier score, calculated as the mean squared difference between predicted probabilities and observed outcomes. Calibration parameters were reported with Wald 95% CI, while a t-based 95% CI was calculated for the mean Brier score. Calibration was visualized using a logistic calibration curve with pointwise 95% CI, plotted against the original TFC probabilities. Observed mortality proportions were also plotted against mean predicted probabilities within quintiles of predicted risk.

Kaplan–Meier survival analysis was performed for the primary outcome of 90-day mortality. Patients were stratified according to the TFC threshold of 10%, and survival curves were compared using the log-rank test [11]. Patients who died within 90 days were considered as events at the recorded time of death, while patients alive at 90 days were censored at 90 days.

As an exploratory analysis, decision curve analysis (DCA) was performed to compare the clinical net benefit of the TFC with that of the CCI alone for the prediction of 90-day mortality. Predicted probabilities were obtained from two separate univariable logistic regression models, one including TFC and one including CCI as continuous predictors. Net benefit was evaluated across clinically relevant threshold probabilities.

The analyses focused on the prognostic association, discrimination, and calibration of the existing TFC in patients admitted to geriatric care. TFC probabilities were calculated using the original formula, without re-estimating its coefficients. No multivariable adjustment for additional clinical predictors was performed. Accordingly, these analyses evaluate the performance of the existing tool in this setting but do not establish its independent prognostic contribution after accounting for other clinical factors.

### 2.7. Ethical Consideration

The study was approved by the local Ethics Committee of the Province of South Tyrol, Italy (approval number: 07-2026). The study was conducted in accordance with the principles of the Declaration of Helsinki.

The requirement for written informed consent was waived by the Ethics Committee because of the retrospective observational design of the study and the inclusion of a frail and clinically complex population.

## 3. Results

All 225 patients meeting the eligibility criteria during the study period were included and analyzed. No eligible patient was excluded because of missing TFC data or unavailable 90-day vital status. The median age was 86 years (IQR 82–90), and the median hospital length of stay was 9 days (IQR 6–13). Overall, 90-day mortality occurred in 62 patients (27.6%), while in-hospital mortality occurred in 26 patients (11.5%).

The descriptive characteristics of the study population are reported in Table 1. Compared with patients alive at 90 days, those who died had a significantly higher burden of comorbidity and frailty, with higher Charlson Comorbidity Index values, more frequent non-autonomy, and more frequent cognitive and motor impairment. TFC values were markedly higher among patients who died within 90 days, whereas age, sex, falls during hospitalization, and hospital length of stay did not differ significantly between groups.

In univariable logistic regression, the TFC was significantly associated with the primary outcome of 90-day mortality. Each 10-percentage-point increase in the TFC was associated with a 43% increase in the odds of death within 90 days (OR 1.43, 95% CI 1.26–1.63; *p* < 0.001). The predicted probability curve showed a progressive increase in 90-day mortality risk, from 9.9% at a TFC value of 0% to 80.6% at a TFC value of 100% (Figure 1). A similar association was observed for in-hospital mortality, with each 10-percentage-point increase in the TFC associated with higher odds of in-hospital death (OR 1.38, 95% CI 1.18–1.62; *p* < 0.001; Figure 1).

For 90-day mortality, the TFC showed an AUROC of 0.747 (95% CI 0.676–0.819), compared with 0.647 (95% CI 0.566–0.728) for the CCI and 0.550 (95% CI 0.467–0.633) for age alone. Paired comparisons demonstrated higher discrimination for the TFC than for the CCI (Holm-adjusted *p* = 0.010) and age alone (Holm-adjusted *p* < 0.001). The difference between the CCI and age was not statistically significant (Holm-adjusted *p* = 0.071).

For in-hospital mortality, the AUROC was 0.732 (95% CI 0.622–0.841) for the TFC, 0.605 (95% CI 0.473–0.737) for the CCI, and 0.543 (95% CI 0.429–0.656) for age alone. The TFC showed higher discrimination than both comparators (*p* = 0.019 for each comparison), whereas the difference between the CCI and age was not statistically significant (*p* = 0.393).

TFC calibration analysis showed a mean predicted 90-day mortality of 30.0%, compared with an observed mortality of 27.6%. The calibration intercept was −0.138 (95% CI −0.483 to 0.207), whereas the calibration slope was 0.658 (95% CI 0.421–0.895), indicating overly extreme predicted probabilities (Figure 2). The Brier score was 0.174 (95% CI 0.144–0.205). The calibration plot showed increasing observed mortality across risk quintiles, with overestimation at higher predicted probabilities (Figure 2).

Using a TFC threshold of ≥10%, the score identified 54 of the 62 patients who died within 90 days, corresponding to a sensitivity of 87.1%. Specificity was 35.6%, positive predictive value was 34.0%, and negative predictive value was 87.9%. Ninety-day mortality was 12.1% among patients with TFC < 10% and 34.0% among those with TFC ≥ 10% (Table 2). Patients with TFC ≥ 10% had significantly higher odds of 90-day mortality compared with those below this threshold (OR 3.73, 95% CI 1.66–8.37; *p* < 0.001). In the exploratory analysis of in-hospital mortality, the TFC ≥ 10% threshold identified 22 of the 26 deaths. Sensitivity was 84.6% (95% CI 65.1–95.6), specificity was 31.2% (95% CI 24.8–38.1), PPV was 13.8% (95% CI 8.9–20.2), and NPV was 93.9% (95% CI 85.2–98.3).

Kaplan–Meier survival analysis showed significantly lower 90-day survival among patients with TFC ≥ 10% compared with those with TFC < 10%. During follow-up, 54 deaths occurred in the TFC ≥ 10% group, compared with 8 deaths in the TFC < 10% group (Figure 3). The survival curves differed significantly at the log-rank test (χ^2^ = 10.27; *p* = 0.001), supporting the association between TFC ≥ 10% and increased 90-day mortality risk (Figure 3).

Finally, DCA was performed to compare the clinical net benefit of the TFC with that of the CCI alone for the prediction of 90-day mortality (Figure 4). Across most clinically relevant threshold probabilities, the TFC showed higher net benefit than the CCI alone, suggesting greater potential clinical utility for mortality risk stratification when comorbidity is integrated with frailty-related domains. At very low threshold probabilities, the net benefit of the CCI was similar to or higher than that of the TFC, whereas at intermediate thresholds the TFC curve remained consistently above the CCI curve (Figure 4).

## 4. Discussion

This study evaluated the performance of the TFC in a specific cohort of older patients admitted from the ED to a geriatric ward. To our knowledge, this is one of the first studies assessing the prognostic value of the TFC in a selected population of older hospitalized patients, rather than in the general ED population. The main finding is that higher TFC values were significantly associated with increased 90-day mortality and in-hospital mortality. Patients who died within 90 days had higher TFC scores compared with patients who survived.

Three findings from this study may be relevant to clinical practice. First, our findings suggest that the TFC may provide useful information not only at the time of ED triage, but also during the transition from the ED to the inpatient ward. The TFC was originally designed as a rapid ED-based tool to identify patients with higher frailty and comorbidity burden [5,6,11]. In this cohort, the score retained prognostic value among older patients who were subsequently admitted to a geriatric ward. The prognostic relevance observed in this cohort suggests that ED-derived TFC information may provide the receiving geriatric team with a structured summary of the patient’s baseline vulnerability. Including this information in clinical handover could support recognition of patients at increased risk; its effects on communication, clinical decisions, and care transitions warrant prospective evaluation.

Second, the TFC may support the early identification of older patients at increased risk of adverse outcomes after ED admission. The score should not be interpreted as a stand-alone tool to determine admission priority or to replace clinical judgment. However, patients with higher TFC values showed a higher risk of adverse mortality outcomes, suggesting that the score may help identify a subgroup of patients who are more complex and potentially more vulnerable during the ED-to-ward transition. This may be particularly relevant in settings characterized by limited resources, reduced bed availability, or prolonged ED boarding, where frail older patients may remain in the ED while waiting for admission to an inpatient ward [14,15,16]. In such contexts, a simple score already available at ED evaluation could help prioritize attention, communication, and early planning of care for patients with higher frailty and comorbidity burden.

Third, this study supports the potential usefulness of an ED-oriented frailty and comorbidity tool even in a purely older population. Frailty assessment in emergency care is often focused on geriatric-specific instruments, such as the Clinical Frailty Scale [2,17]. These tools remain important, particularly for comprehensive geriatric assessment and inpatient evaluation. However, the ED is a heterogeneous setting, where vulnerability is not limited to age alone [4,6]. Patients may present with complex comorbidities, disability, chronic functional limitations, cognitive impairment, or recent hospitalizations. The TFC was developed to capture this broader background vulnerability by combining comorbidity and frailty-related domains [6,11]. Although this mixed structure may be considered less specific than purely geriatric frailty scales, it may represent a pragmatic advantage in the ED context, where rapid and standardized risk stratification is needed [18]. Our results suggest that this broader approach remains informative even when applied specifically to older patients admitted to geriatric care.

Importantly, these findings do not imply that the TFC should replace more detailed frailty assessments after hospital admission. Rather, the TFC may be considered an early screening and communication tool. Information collected at ED triage may help inpatient teams identify patients who could benefit from closer monitoring, early multidisciplinary evaluation, or more structured geriatric assessment after admission. Therefore, the potential clinical value of the TFC lies not in substituting existing geriatric tools, but in improving continuity of information between the ED and the ward.

The prognostic performance of the TFC was clinically relevant. The AUROC values for both 90-day mortality and in-hospital mortality indicated moderate discriminatory ability. The prespecified ≥10% threshold identified most patients who died within 90 days, but its limited specificity and PPV support its interpretation as a screening signal for further assessment rather than a stand-alone prediction of death. Mortality below the threshold remained 12.1%, indicating that a negative classification does not exclude clinically relevant risk. The TFC retained prognostic relevance and moderate discrimination in this selected geriatric population. The calibration slope below 1 indicates that the original probabilities were overly extreme, identifying a potential target for refinement when applying the tool to geriatric inpatients. Recalibration, followed by validation in additional cohorts, could improve the accuracy of absolute risk estimates, building on the prognostic information already captured by the score.

The exploratory DCA should be interpreted in light of the established role of the CCI in geriatric risk stratification. The CCI was originally developed as a prognostic comorbidity index to estimate mortality risk related to chronic disease burden and remains one of the most widely used morbidity measures in older hospitalized patients [10,19]. Previous studies in acutely hospitalized older adults have shown that higher CCI values are associated with increased short- and long-term mortality, including 3-month, 1-year, and 5-year mortality [20]. Similarly, in older patients admitted through the ED, CCI has been reported to predict mortality, cardiovascular mortality, rehospitalization, and length of stay [21]. However, comorbidity alone may not fully capture the multidimensional vulnerability of geriatric patients. Recent evidence suggests that the CCI is frequently used and prognostically relevant, but also that other morbidity or geriatric multidimensional measures may outperform the CCI for mortality prediction in older inpatients [19]. In this context, the TFC may offer a clinically meaningful extension of comorbidity assessment because it integrates the CCI with frailty-related domains. The TFC combines weighted information on comorbidity and frailty-related domains into a composite prognostic risk marker. This integrated assessment supports the identification of different levels of mortality risk within the geriatric inpatient population, while the calibration findings indicate that its original probability estimates may require recalibration in this setting. The exploratory DCA suggested that the model using the TFC offered a more favorable balance between correctly identifying patients who died and falsely classifying survivors as high risk than the model using the CCI alone, at intermediate threshold probabilities. Net benefit weights false positive classifications according to the chosen decision threshold. The comparison also includes selecting all patients or no patients for an additional clinical action, such as further assessment. These findings suggest potential value for targeting clinical attention, without establishing that TFC-guided care improves patient outcomes.

The Kaplan–Meier analysis further supported the prognostic relevance of the TFC threshold. Patients with TFC ≥ 10% had significantly lower 90-day survival compared with those with TFC < 10%. This finding reinforces the association between higher baseline frailty and comorbidity burden and worse short-term outcomes after hospital admission. However, the present study cannot demonstrate that faster admission, different bed allocation, or TFC-guided interventions would reduce mortality. These aspects should be evaluated in future prospective studies.

This study has several limitations. First, its retrospective, single-center design limits the generalizability of the findings. Second, the sample size was limited, especially for the secondary outcome of in-hospital mortality. Third, detailed data on acute illness severity at ED presentation were not available. The analyses were not adjusted for sex, admission diagnosis, acute illness severity, or treatment limitations. Consequently, the observed association between TFC and mortality does not establish an independent prognostic contribution after accounting for these factors. Patients with similar TFC values may have different mortality risks depending on their acute condition and treatment goals. Future studies should evaluate the contribution of TFC alongside these clinical predictors. The original ED scores and a component-level record of corrections were not available for comparison. We could therefore neither quantify the frequency and nature of corrections nor repeat the analyses using the original ED values. Verification also incorporated inpatient documentation and was not blinded to in-hospital mortality, representing a potential source of information bias, particularly for the secondary outcome. Finally, this evaluation involved investigators who participated in TFC development. Further assessment by independent research groups would strengthen the evidence on its performance.

## 5. Conclusions

Among older patients admitted directly from the ED to a geriatric ward, higher TFC values were associated with increased 90-day and in-hospital mortality. Discrimination was moderate, and the prespecified ≥10% threshold identified most 90-day deaths, although specificity and PPV were limited. These findings indicate that information collected at ED presentation remains prognostically informative within this selected geriatric population. Prospective impact studies are needed to determine whether incorporating this information into geriatric care provides benefits beyond prognostic stratification.

## Figures and Tables

**Figure 1:**
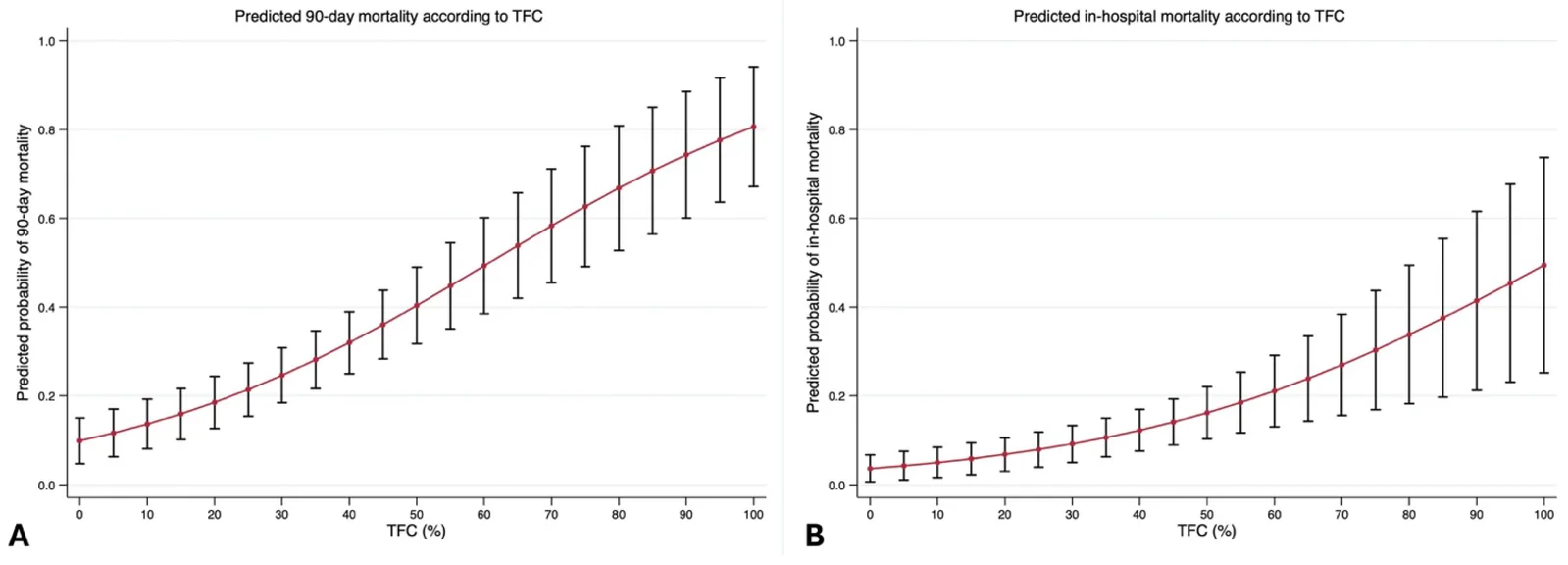
The predicted probability of mortality according to TFC values. Predicted probability curves derived from univariable logistic regression models showing the association between the TFC and mortality outcomes. Panel (**A**) shows the predicted probability of 90-day mortality, while Panel (**B**) shows the predicted probability of in-hospital mortality. Higher TFC values were associated with a progressive increase in predicted mortality risk.

**Figure 2:**
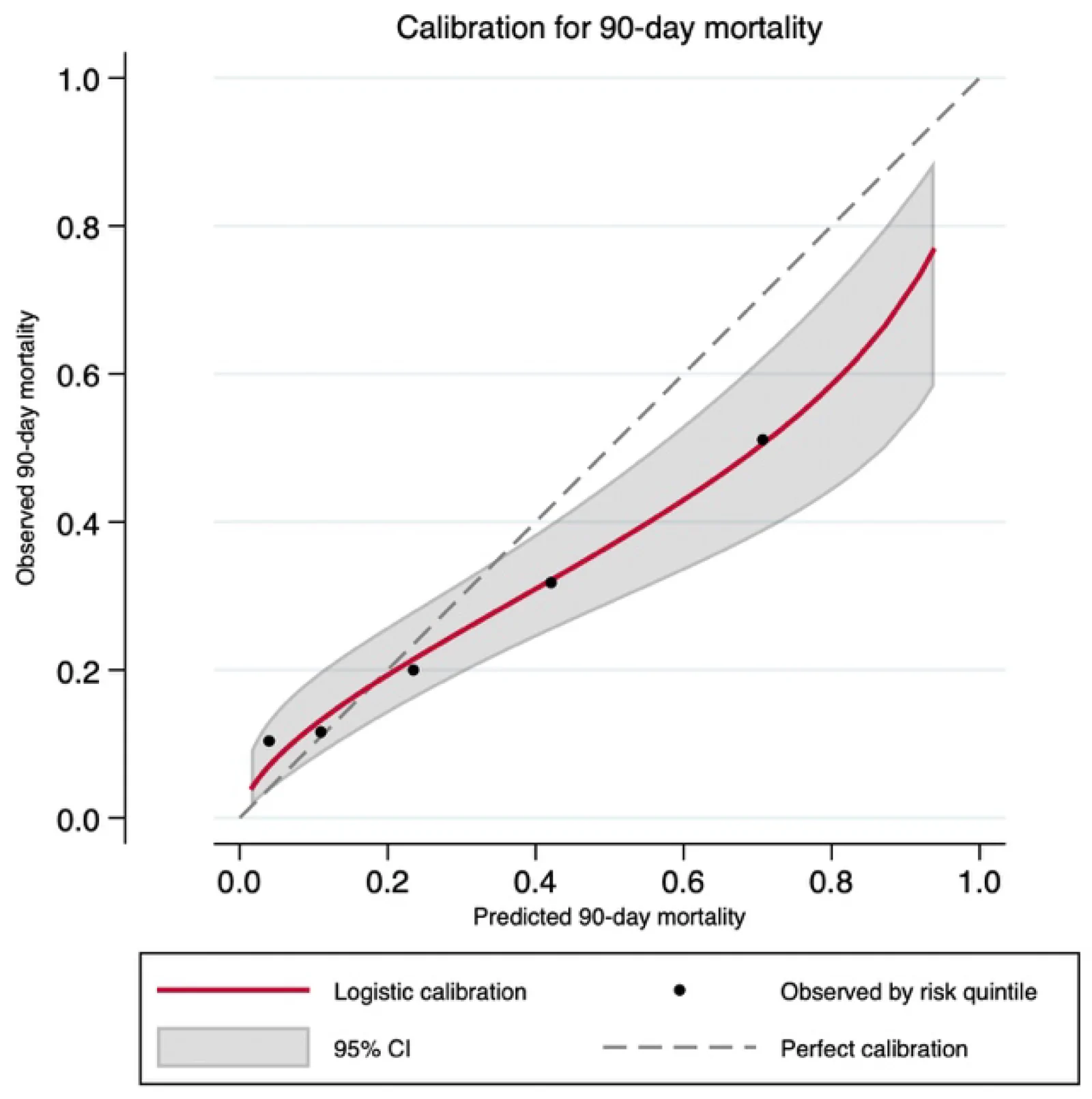
Calibration of the TFC model for 90-day mortality. The solid red line represents the logistic calibration curve, with its pointwise 95% confidence band. Points represent the observed mortality proportion plotted against the mean predicted probability within each risk quintile. The dashed diagonal indicates perfect calibration.

**Figure 3:**
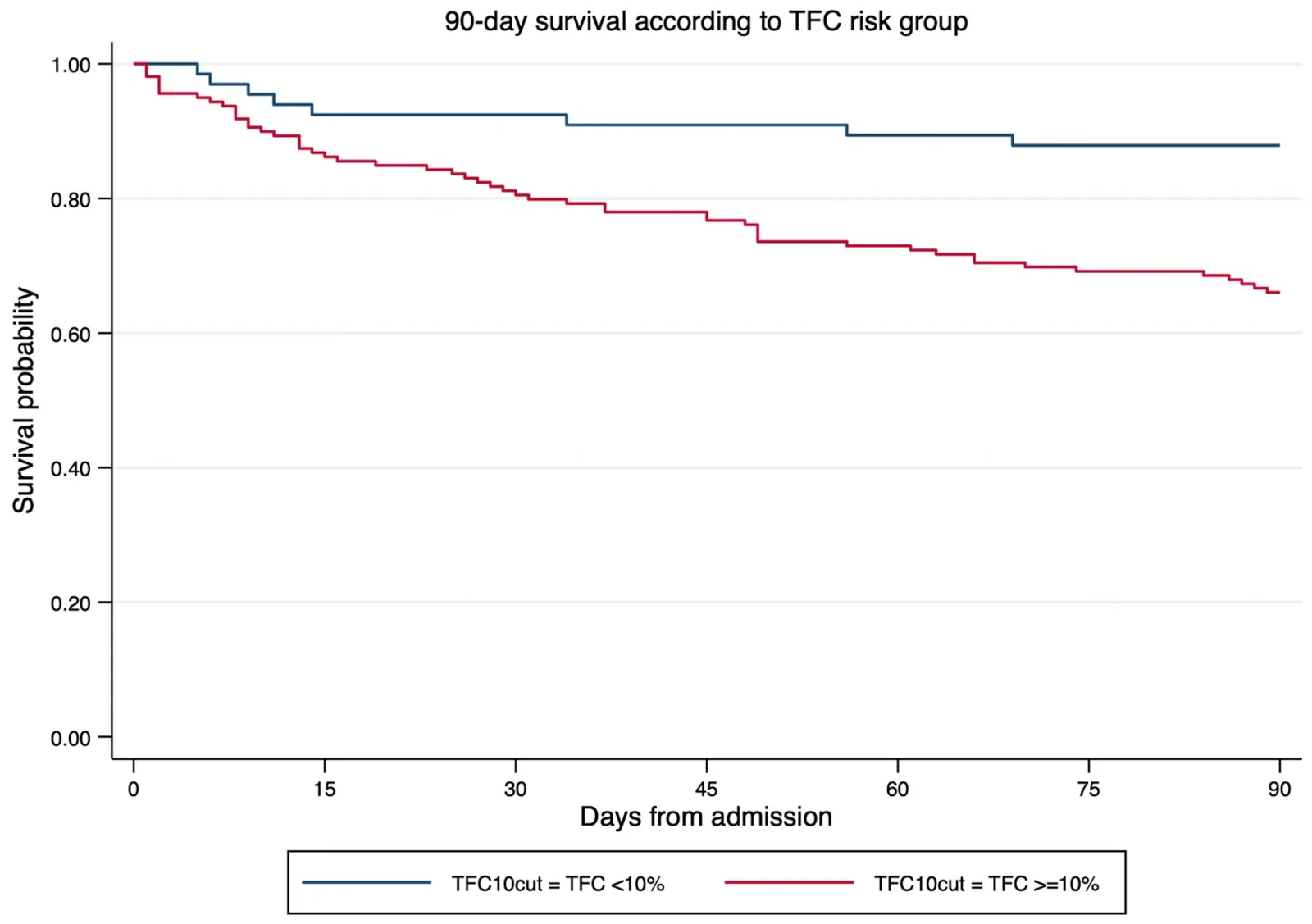
Kaplan–Meier analysis of 90-day survival according to TFC risk group.

**Figure 4:**
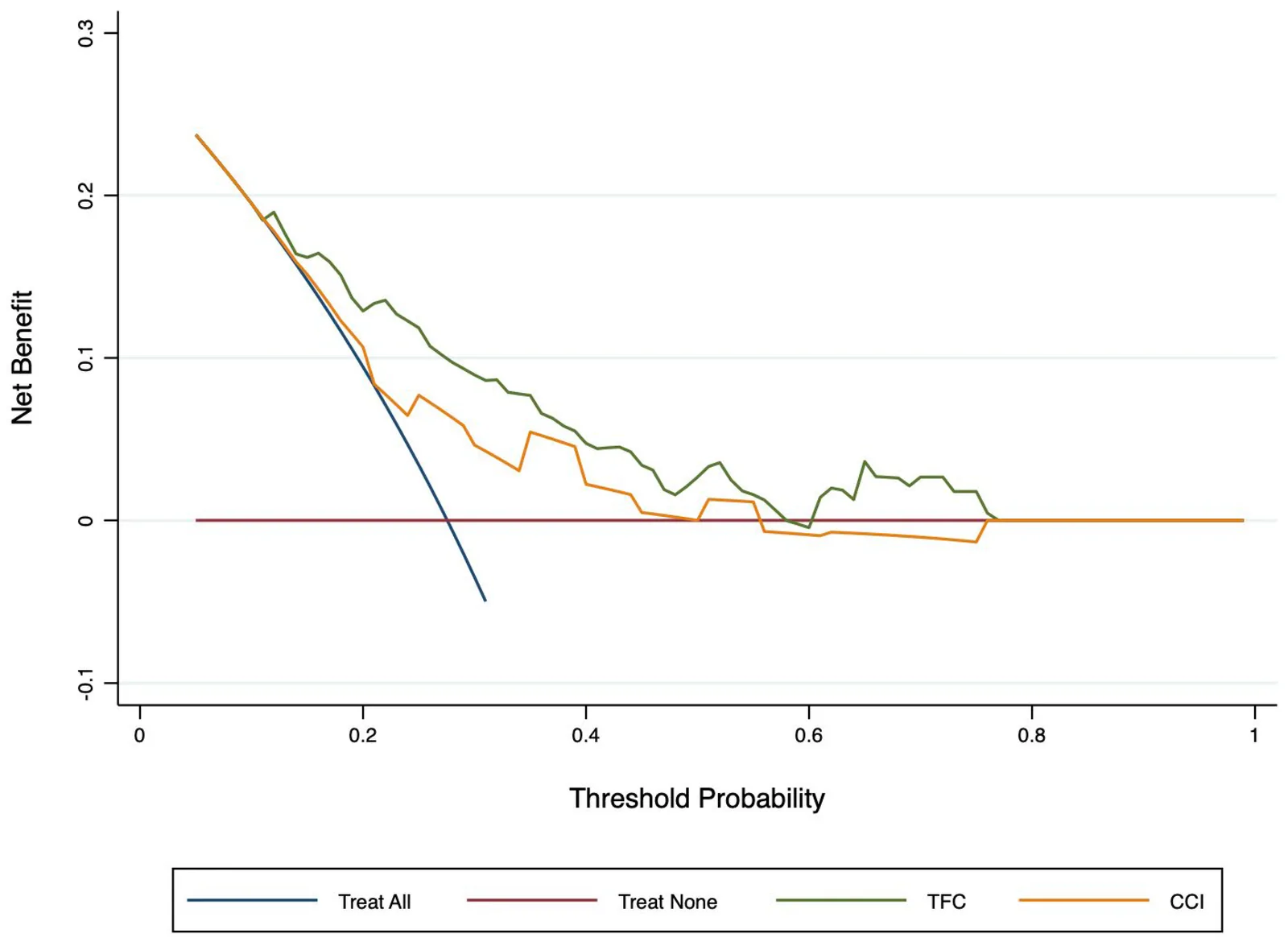
DCA comparing the TFC and the CCI for 90-day mortality. Decision curve analysis comparing the net benefit of the TFC and the CCI alone for the prediction of 90-day mortality. Threshold probability represents the risk level at which a patient would be considered high risk. Net benefit balances true positive identification against false positive classification; therefore, the curve with the highest net benefit at a given threshold indicates the strategy with the greatest potential clinical utility. The TFC showed higher net benefit than the CCI alone across a broad range of intermediate threshold probabilities. Treat-all and treat-none strategies are shown as reference curves.

**Table 1:** Baseline characteristics of study population according to 90-day mortality status.

Variables	Alive at 90 Days	Died Within 90 Days	*p*-Value
Patients, n (%)	163 (72.4)	62 (27.6)	
Sex, n (%)			0.194
Female	92 (56.4)	29 (46.8)	
Male	71 (43.5)	33 (53.2)	
Age in years, median (IQR)	86 (81–90)	87 (83–91)	0.417
Arrival mode to the ED, n (%)			0.062
Self-arrival	55 (33.7)	13 (21.0)	
Emergency services	108 (66.3)	49 (79.0)	
Charlson Comorbidity Index, median (IQR)	7 (5–8)	8 (6–10)	<0.001
Not autonomous in chronic condition, n (%)	82 (50.3)	50 (80.6)	<0.001
Altered cognitive status, n (%)			<0.001
No	102 (62.6)	24 (38.7)	
Mild	56 (34.3)	21 (33.9)	
Severe	5 (3.1)	17 (27.4)	
Altered motor condition, n (%)			0.001
No	81 (49.7)	18 (29.0)	
Uses an aid but is not bedridden	75 (46.0)	33 (53.2)	
Bedridden	7 (4.3)	11 (17.7)	
Falls during hospitalization, n (%)	5 (3.0)	1 (1.6)	0.545
Hospital LOS, days, median (IQR)	9 (6–13)	11 (6–14)	0.192
Discharge destination, n (%)			<0.001
Home	139 (85.3)	20 (32.2)	
Nursing home, previous resident	10 (6.1)	6 (9.7)	
Nursing home, new admission	5 (3.1)	3 (4.8)	
Post-acute care facility	9 (5.5)	7 (11.3)	
Death during hospitalization	0 (0.0)	26 (41.9)	
TFC, %, median (IQR)	19.4 (6.5–43.8)	49.5 (31.4–72.3)	<0.001

**Table 2:** Prognostic performance metrics of the TFC ≥10% threshold for 90-day mortality.

	Alive After 90 Days	Dead After 90 Days	Total
TFC < 10%	58	8	66
TFC ≥ 10%	105	54	159
Total	163	62	225
Sensitivity: 87.1 (76.1–94.3) Specificity: 35.6 (28.3–43.4) PPV: 34.0 (26.6–41.9) NPV: 87.9 (77.5–94.6)			

## Data Availability

The data supporting the findings of this study are available from the corresponding author upon reasonable request, subject to the necessary institutional and ethical authorizations. The data are not publicly available because they contain patient-level clinical information, and public dissemination is restricted by patient confidentiality requirements and the conditions of the Ethics Committee approval.
